# Anchylostamum Duodenale, with Report of Cases

**Published:** 1897-03

**Authors:** F. G. Möhlau

**Affiliations:** Buffalo, N. Y. 1298 Jefferson Street


### ANCHYLOSTAMUM DUODENALE, WITH REPORT OF CASES.

By F. G. MÖHLAU, M. D., Buffalo, N. Y.

BEFORE referring to the cases of this disease that recently came under my observation, I deem it not out of place to give a short outline of the peculiarities of this parasite. The study of this nematode is of the utmost importance in medical practice, on account of the great destruction it is apt to produce by leeching out the blood of the intestinal tract when once it has therein gained entrance. It resembles the oxyuris vermicularis, but is still more like the trichinæ intestinalis. One parasite, the anguillulum intestinalis, not infrequently occupies the small intestines alongside with the anchylostamum. Their resemblance is great, their eggs are much alike, and therefore require close study. For their difference I would refer to the text-books. It has a round body, resembling that of the worms mentioned, the head-end has a mouth-like process, which is armed with chitin teeth. To this the tractus intestinalis is attached. The length of the parasite is varying, the male being smaller than the female ; the eggs are oval in shape, also varying in size, the average measure being about .03 mm. in size and covered with a fine shell. The process of segmentation is permanently going on, and is very easily watched, especially so if the feces are placed in an even and warm temperature.

The picture of the disease produced by the parasite has been described as early as 3,450 years ago, in a work of Papyros Ebers by name. The parasite itself was discovered by Angelo Dubini, in Milan, in 1838. Von Sieboldt gives a good description of it. Somewhat later, in the year 1851, it was recognised by Von Griesinger as the true cause of the Egyptian chlorosis. Everywhere we find reference made to this parasite whenever there is spoken of epidemics of chlorosis, principally if we hear of such epidemics in southern or warm climates. The finding of several cases in a very short time makes me think that the disease is occasionally found in northern states. It is not as rare as spoken of in Dr. Pepper's text-book of Theory and Practice of Medicine, 1895, Vol. II., page 883. He writes : "So far, no observations have been made in this country, but it is possible the disease exists in some of the Southern States." The not finding of the parasite in Northern America seems to me rather due to the infrequency of chemical and microscopical examination of feces by the general practitioner. If laborers are afflicted with the disease while in Europe, how can it disappear while coming over the ocean ? The symptoms and etiology of this infection seem to differ with the country in which it is referred to. In every journal and text-book we find decided differences as to the picture of the disease the parasite is apt to produce. Certain trades seem to be more afflicted than others. Certainly we find more frequent mention of this disease after the building of the St. Gotthardt tunnel. Shortly after that time it became epidemic in various parts of Europe. Men employed in brickyards, miners and potmakers seemed to be the principal sufferers. It is a fact that after the building of the new tunnel a greater mixing up of those laborers took place, and it is natural the miners and men working in brickyards took to the notion of traveling, and they imported the disease to America.

*The Mode of Infection.*—The development of the egg after it has left the body deserves some mention. After it has left the body it soon sets the embryo free, which leaves the shell as a rhabditis forms, which soon encysts itself, and these larvæ are apt to resist all kinds of weather and temperature. Infection may be brought about in many a way, as, for instance, after working in clay the hands may be infected with the larvæ. In summer season it is proved by the experiments of Von Schlopp that the larvæ may be taken up by dust, carried by the wind and thus swallowed, thereby reaching the digestive tract, and producing symptoms more or less severe when developed to the mature worm. Children are very easily infected, because they have a decided notion or preference for play in mud or clay piles, and after making mud pies frequently consider a piece of such pie a delicious morsel. Last, but not least, we have to consider a very important way of infection, that is, by eating vegetables grown in infected soil,—ground berries, strawberries, salads, lettuce, celery,—in fact all that have grown on low soil and in the ground directly. After the larvæ have reached the stomach, where no action is exerted upon them, they pass on to the small intestines, until the alkaline juice below the common bile-duct dissolves the thin cover, and the fully-developed young worm is set free. As far down as the jejunum the parasites may be found. Very seldom the worm is found in the large intestines. In the small intestines these nematodes attach themselves like leeches to the mucous membrane, boring their way as far as the submucosa, there holding firm with their hook-like teeth, and by the leeching movements the blood is taken up.

The worm seems to make use of the fluid part of the blood only, as one can observe it expelling the red cells through the anal opening, having it freed from its fluid contents. This shows that only sanguine fluid is necessary for the parasite to live on. By means of the microscope the process is easily observed how the red cells leave the tractus intestinalis without any structural change, only in a somewhat shrunken condition. The symptoms of the disease seem to be manifold : if but few of the nematodes are present, no symptoms may appear, providing the individual is otherwise healthy and of strong constitution ; as soon as development of a greater number of these parasites takes place, the symptoms may be aggravated in form. The female takes up more nourishment than the male. As the loss of blood increases, the more pronounced becomes the anemia, the characteristic trouble produced by this intestinal parasite. A patient afflicted with the disease resembles the American Indian in color, perhaps more nearly the Esquimau. The blood seems to be poor in coloring matter and of watery consistence ; in fact, if the nematodes exist in large numbers, say several hundred, the progressive anemia becomes more marked, until we observe the perfect picture of a progressive pernicious anemia, and thus a fatal termination is not excluded. Diagnosticating a case of pernicious anemia, the anchylostamum duodenale ought at once to be looked for. The anemia may be produced in various ways ; in the first place, by the irritation of these worms exerted upon the intestines ; furthermore, there may be absorption of certain toxins produced by the worm and absorbed by the intestinal mucous membrane ; also, the excreta of the parasite in general being absorbed, really the absorption of the excreta and secreta must be the greater factor in bringing about the early symptoms of such anemia.

Very naturally, these toxins are taken up by the blood stream, with the food being digested in the intestinal tract; but there seems a special toxin manufactured by the nematode exerting a peculiar influence over the blood and its circulation. Whenever there is a worm falling off, more or less hemorrhage takes place on that part. Several patients often felt a sensation of warmth, caused by the flowing of blood in the intestines, especially in the morning, and express the sensation as being not unlike the nose bleeding. After such complaint I always found blood in the feces. No matter how small the hemorrhage, it does not fail to influence the system. At post mortem, of which I saw one while in Berlin, many brownish spots are found, which are the remains of the attachment of such a worm. The influence of the worm upon the digestive tract is apt to produce severe diarrhea, at times accompanied by vomiting ; in fact, it may resemble cholera nostras in every particular, often going so far as to bring a patient to a state of collapse. There may follow complete rest and normal function for a period, then again starting up the same symptoms in aggravated form. A period of longer or shorter duration, even one or two years, where hardly any abnormal symptoms appear, is not infrequent.

On the part of the patient, the want of oxygen seems increased, and the waste products as $C.O_2$ seem to be given off in excessive amount. The oxydisation of all food-stuffs seems to be accelerated, yet with little benefit to the patient. There seems to be an auto-intoxication going on all the while in the intestinal tract, and in this way we find causes for the various stages of anemia. The appetite of the patient often is intense, *i. e.*, it seems that he cannot eat sufficient ; then, again, periods of longer duration and the patient is deprived of all appetite for food. Thirst may be very intense ; the heart is influenced on account of the toxins present. The blood is the part that suffers most, and this may be in any condition, according to the severity of the disease.

### SYMPTOMS.

I find the symptoms in this disease to be characteristic in nearly all cases, as the peculiar expression of the patient, the anemia, the diarrhea, always preceded by a severe attack of colic, the pain, as a rule, being on the left of the median line ; in fact, it seems to be right over the pancreas, while it is just beneath common bile-duct in the upper part of the small intestines. Pruritus ani is intense, especially in the evening ; at night it may become so intense as to awaken the patient. Of peculiar notice is the difficulty of hearing, due to the anemia, which subsides as the other symptoms of the disease decrease. A one-sided headache, which I have observed to be most intense at night, is relieved when some food is taken, and therefore seems mostly due to the empty stomach ; yet there is more or less headache all the time, and it is an obstinate symptom to combat. All the pain seems to come on mild in the beginning, increasing in intensity until it has reached its climax, and then subsiding in the same way as it came. I have also noticed a desire to swallow accompanying the trouble, not unlike the swallowing of the globus hystericus. Furthermore, there is, characteristic of this, a swelling nearly over the pancreas. When first noticed, it seems like a tumor, which decreases in size as soon as cathartics are administered and have taken effect. Now, with the aid of the microscope, the diagnosis is easy, as not only the eggs but the worms are found in the stools. The finding of the parasite and its eggs naturally confirms the diagnosis. I am sure that many cases of anchylostamum have been mistaken for cholera nostras, or as being due to some unknown poison, or other cause, as I have found out myself in several instances. I had five cases under observation, which I had gathered in a comparatively short time, which makes me believe that the parasite has a home in Buffalo, where there are a number of inhabitants of Italian and Polish laborers and clay soil in and around the city, both of which seem advantageous to the existence of the nematodes and their distribution.

Case I. Mr. R., aged 48, came to me in November for treatment. His expression peculiar, which I could describe as an expression of fear, complaining of feeling very weak and dizzy. Patient says that he has felt sickly during the last three years. Had been working in a brick-yard several years ago. His skin is bronze colored; mucous membranes all show signs of severe anemia. His appetite is very varying, but mostly poor. His bowels are usually constipated, and at times has diarrhea, with severe pain. His circulation is very poor, vessels seem relaxed. Has been treated at various times for anemia, and also for liver complaint. Physical examination : I found systolic murmurs over heart ; the heart's action weak ; the pulse irregular, slow, intermittent ; respiration shallow, but labored; the blood seems watery, the number of red corpuscles decreased ; the number of red cells to the cubic millimeter decreased about one-half million ; the stomach normal in size, tender to touch ; liver seems normal, but also very tender ; the pancreas and small intestines are extremely painful to touch and considerable swelling is noticed. Patient had difficulty in walking for a while ; his memory seemed somewhat weakened ; digestion is very poor ; after having administered a cathartic, examination shows food barely digested—undigested starch in abundance and considerable amount of undigested meat fiber; large numbers of eggs of dochimus duodenalis; kidneys somewhat inactive and a trace of albumin.

Treatment for the parasite causes improvement of the patient. The improvement is steady, and the patient is able to go to work again, something he has not been able to do during the last year.

CASE II. Albert H., aged 7, had an attack of pneumonia and an attack of typhoid fever. Recovery was unusually protracted. Examination of the stools, after a mild cathartic, revealed the presence of a large number of the eggs of dochimus duodenalis, and an excessive number of trichomonas intestinalis and cercomonas intestinalis.

Treatment for the disease brought about a rapid recovery. The change of the blood was that of progressive anemia. Infection was traced to one Polish and two Italian laborers who were infected with the disease while employed in the brickyard of the patient's grandfather, the playground of the children.

CASE III. Mr. S. O. came to me September 14th for treatment, a second time since 1894. He is 52 years of age, a farmer. He was treated for pernicious anemia several times without any marked success. His complexion is bronze, his look unsteady; his pulse is slow and weak, his temperature 100 2-10, respiration 26; the white of the eyes show marked yellow, his blood-vessels seem enlarged, but not thickened; capillary circulation fair; anemic murmurs to be heard in the heart sounds. Examination of blood: poikilocytosis marked, red cells decreased in number; digestive apparatus in very poor condition, ulcerations of the rectum extend up as far as 4-6 inches. Stomach contents: rare meats, well digested; starches, barely digested; stomach not enlarged, the movements somewhat hastened; vomiting quite frequent. Microscopic examination of the feces: greater number of parasites, red cells; leucocytes, amebæ coli and eggs of dochimus duodenalis in abundance; furthermore, epethelial cells, triple phosphates, and principally trichomonas intestinales and cercomonas intestinalis, are present; tongue, pale, heavy-coated; liver, normal; spleen, somewhat enlarged; skin has a whitish-yellow appearance; the kidneys, somewhat irregular in action. Patient was infected with syphilis eleven years ago, and, furthermore, committed self-abuse until late years; his mental condition is somewhat deranged, and evidences of progressive spinal trouble seemed present. Examination of urine: normal, but faint trace of albumin. September 14th, at 9 P. M., the patient had an attack which resembled cholera nostras; temperature, 103½; pulse, 120; breathing, 28 to the minute. All ordinary remedies failed to check it, until 4 o'clock in the morning, when the old French remedy of claret boiled with a considerable amount of cinnamon checked the attack. About 9 o'clock a full picture of typhoid fever seemed to have developed ; temperature, 103 8-10 ; pulse, 114 ; respiration, 26 ; typhoid spots on lower chest and abdomen ; tongue, thick brown coating, and ilio cecal region very tender ; patient himself very drowsy and hard to awaken. September 17th—The symptoms remain the same. Commenced to give sponge baths at short intervals. Stimulants : lactophenin, in eight-grain doses, if required. September 18th—Symptoms remain the same, until the 26th, when all typhoid symptoms seem to have disappeared all at once. High rectal douches bring about free evacuation ; great number of the parasite and eggs in the discharge. From that day the patient improved, but very slowly, always complaining about pain over his pancreas. Treatment: douches continued, mild vermifuges, and bowels kept regular ; anemia remains the same, progressive, pernicious anemia ; all remedies fail. The number of the parasites became less, so also the eggs. On October 12th no eggs nor parasites can be found ; patient begins to feel better in every respect and leaves for home, this being the second time he underwent the worm cure. Examination of blood before departure showed marked improvement.

CASE IV. Frank R., German, aged 46, stonecutter, married, family history negative, came for treatment September 3d. Patient had been well until six years ago, when he had an attack of gonorrhea; evidences of chronic symptoms. At that time he had orchitis epididimitis. Three months ago he had a mild sunstroke, at least that is what he was treated for. *Status presens :* Patient has a brownish color, signs of severe anemia; breathing is difficult; asthmatic ; heart, systolic murmurs, very weak ; pulse, small and frequent ; patient complains of permanent tired feeling ; digestion and appetite very poor ; complains of having hemorrhages from the bowels at intervals ; on examination, no hemorrhoids ; rectum free from all disease ; cough, short and hacking, no expectoration, patient having a desire to swallow frequently ; kidneys, slightly irregular ; urine, normal ; skin, muddy color ; liver, enlarged, also spleen ; genitals, chronic gonorrhea ; blood, red elements decreased 3½ millions red cells, irregular in size and shape. Examination of feces : eggs of anchylostamum duodenale, also eggs of tenia solium ; foodstuffs badly digested.

The patient has been working in a brickyard along with Italians, who oftentimes suffered with severe colic, of which he now complains himself at times, and diarrhea.

CASE V. Antonia F., bricklayer, aged 58, has been employed at the building of the St. Gotthard ; also very anemic ; history same as before.

Both patients improved in every respect on antiparasitic treatment.

1298 JEFFERSON STREET.

